# Joining forces: the need to combine science and ethics to address problems of validity and translation in neuropsychiatry research using animal models

**DOI:** 10.1186/s13010-019-0085-4

**Published:** 2020-01-23

**Authors:** Franck L. B. Meijboom, Elzbieta Kostrzewa, Cathalijn H. C. Leenaars

**Affiliations:** 1 Ethiek Instituut, Universiteit Utrecht, Yalelaan 2, 3584 CM Utrecht, The Netherlands; 2 Faculty of Veterinary Medicine, Universiteit Utrecht, Yalelaan 2, 3584 CM Utrecht, The Netherlands; 3 SYRCLE, Radboud University Medical Center, Nijmegen, The Netherlands; 4 Institute for Laboratory Animal Science, Hannover Medical School, Hannover, Germany

**Keywords:** Neuropsychiatric disorders, Animal models, Validity, Applied research, Translation, Ethics

## Abstract

**Background:**

Current policies regulating the use of animals for scientific purposes are based on balancing between potential gain of knowledge and suffering of animals used in experimentation. The balancing process is complicated, on the one hand by plurality of views on our duties towards animals, and on the other hand by more recent discussions on uncertainty in the probability of reaching the final aim of the research and problems of translational failure.

**Methods:**

The study combines ethical analysis based on a literature review with neuropsychiatry-related preclinical research as a case study.

**Results:**

Based on the analysis and the case study we show that neuropsychiatry-related preclinical research is an especially interesting case from an ethical perspective. The 3R principles (Replacement, Reduction and Refinement) are used to minimize the negative consequences for the animals used in research. However, neuropsychiatric research is characterized by specific challenges in assessing the probability of success of reaching the final aim, due to our limited mechanistic knowledge of human neuropsychiatric illness. Consequently, the translational value of the currently used animal models may be difficult to prove, which undermines the validity of these models and complicated the ethical assessment.

**Conclusions:**

We conclude that a combined approach that deals with both science and the ethical dimensions is necessary to address the problems of validity and translation in neuropsychiatry-related preclinical research. We suggest this approach to comprise first, improved experimental methods, e.g. by using systematic reviews, second, a more patients-based approach that leads to models that reflect interindividual variation better, and third, more interdisciplinary cooperation.

## Background

According to current European laws and policies on the use of animals for scientific purposes, animal experimentation is considered ethically acceptable only if it delivers knowledge that weighs up against the suffering of the animals used (EU 2010 [1, 2];). However, to make such an assessment is not easy. Debates on the ethical acceptability of animals in research are characterized by plurality and disagreement [3]. This disagreement finds its origin in different views on the moral position of animals and the value of the aims of the research, but is also due to problems of probability and uncertainty. Each of these aspects have always been complicating factors for an ethical assessment. Research with animals is evaluated before the actual experiment takes place. Therefore, one never can be completely certain about the question whether the direct or final aim will be reached [4]. This situation has been further complicated by more recent discussions about the quality of the research models used at the translational success of preclinical animal research [5–9].

The aim of this paper is to present and analyse animal use for neuropsychiatry-related research as a case study to show how questions about the value of the animal models used further complicate the ethical assessments. Our reflection consists of three parts. First, we present the background and increasing complexity of the ethical debate on animal research. Second, we show why neuropsychiatry-related research is an interesting case from an ethical perspective. Finally, we aim to show that, both from a normative and a scientific perspective, research quality benefits from ethical reflection.

### The ongoing ethical debate: animals as moral subjects

Ethical deliberations in the context of animal research often start with the question whether we should treat animals as moral subjects. If so, animals should be taken into account in our moral reasoning for their own sake. In animal research, we take the interests of the animals into account, as good animal health and welfare can also benefit the research. However, considering animals as moral subjects is taking another step. This moral standing implies that one has direct reasons to take the interests of animals into account rather than only because it coincides or correlates with human interests. Acknowledging that animals are entities that have moral status entails direct implications for our duties towards them. However, even if we agree on *that* (some) animals have moral status, there is still debate on *what* it implies in terms of our duties. Some argue that it is morally wrong to take the life of an animal for any reason. Others stress that the most important duty is to prevent suffering. These differences have their origin in the *why* question, i.e., the various arguments that underlie the claim of animals having moral standing, such as sentience, ability to suffer, higher cognitive abilities, capacity to flourish, sociability and animals being “subjects-of-life” [10–13].

Before jumping to the conclusion that there seems to be consensus about the moral position of animals, we note that some expressed clear arguments against the idea of animals having a moral status. They claim that humans do not have any direct moral obligations toward other animals. The arguments for this position are diverse, but some of them refer to the superiority of the human species. The notion that there are empirical differences between species is widely acknowledged, but the superiority view emphasizes the differences between species as morally relevant and as affecting the moral status of humans and other species. This results often in the idea that human preferences are more important than those of other species for the only reason that humans are more important as a species. Without additional morally relevant arguments, this position is flawed, and referred to as ‘speciesism’ [14]. Singer argues that this position is like racism or sexism, which have been proven to be flawed, as they directly derive normative arguments from empirical differences. Likewise, stressing the empirical difference between humans and non-human animals cannot be the only argument to settle ethical questions of animal use.

Others contrived additional arguments to substantiate the moral difference between animals and humans (cf. [15]). They emphasize that humans are superior to animals in terms of rationality, ability to communicate, and self-awareness. Consequently, they argue that animals cannot count independently in our moral reasoning. Referring to superior human rationality or moral autonomy is, however, not beyond debate. Authors such as Tom Regan, Peter Singer and Richard Ryder show the complexity of the discussion by introducing the so-called Argument from Marginal Cases [16] and more recently Horta [17] used the Argument from Species Overlap. Although human infants and intellectually disabled people may not meet all the cognitive criteria essential to be acknowledged as moral agents, we nonetheless commonly agree that we can have duties towards them, and that it would be morally wrong to conduct harmful experiments on them. If one refers to human rationality as the necessary criterion to enter the moral circle, we still need, out of consistency, additional moral arguments of why we are allowed to experiment on (non-rational) animals.

For this moment, we can conclude that despite the plurality of views, there are strong reasons to take animals into account in our moral reasoning for their own sake, which is also reflected in European and national (e.g. in the Netherlands) legislation [18].

### Ethical assessment of animal testing: a complex task further complicated

If one acknowledges that sentient animals have moral status, this does not immediately imply that one disagrees with the use of animals in experimentation in any situation. Some lines of reasoning do lead to an abolitionist position, but not all ethical positions that acknowledge that animals have moral status exclude justification of using animals for research [18]. The most common argument to justify the use of animals in research is the expected benefit to humans, but also to animals themselves in veterinary practice. Regardless of the anticipated benefits, replacement, reduction and refinement (the 3R principles, [19]) are used to minimize the negative consequences. The justification based on expected benefit can also be recognized in the EU directive (EU 2010) that requires a harm-benefit analysis (HBA) for each animal experiment and starts from the assumption that an experiment can only be justified if the expected harm is weighed against the expected benefits. Based on the situation of ongoing animal research one may conclude that that many studies directly or ultimately have important aims. However, the situation is far more complex. First, making a HBA including the assessment of the potential benefits is not easy. There are a number of difficulties related to the aim and the procedure of the analysis. These comprise, for instance, securing transparency in the process and the level of consistency between outcomes of the analysis, while at the same time still allowing room for the dynamics of ethical deliberation [20]. Furthermore, members of ethical committees are struggling with this task themselves. They often tend to focus on the technical issues, on which one assumes to reach consensus more easily rather than on the ethical questions whether the benefits of the research exceed the expected harm to the animals (cf. [21]). Second, the complexity on a procedural and practical level can partly be explained by plurality on a theoretical level. The diversity of views cannot be reduced to the textbook distinction between consequentialist and deontological approaches [22]. The Nuffield Council [3] also shows that the plurality of theories results in a continuum views, rather than in principled pro or contra positions. Many other approaches, including virtue ethics, care ethics and pragmatism can analyse and deal with moral conflicts between human health and wellbeing and animal pain and suffering. However, they do so in different ways and with different practical consequences. It makes a substantial difference whether the ethical assessment is framed in terms of human and animal welfare or whether it is perceived as a conflict between duties of care in which relations between humans and animals play an important role. In the latter approach, the fact that dogs are often perceived as closer to humans than pigs can be a relevant argument in the assessment, where in a welfare-only approach this would be considered as irrelevant for the moral justification.

However, the third aspect that complicates the ethical assessment is a challenge for a broad range of ethical theories. Each theory that considers animal testing as a moral problem and therefore requires some sort of moral justification has to deal with uncertainty; the uncertainty of the outcome of an experiment, and if it will contribute to its final aim. Therefore, for the ethical justification of research with sentient animals we need to determine the extent to which the use of an animal model delivers useful outcomes, and if it is an efficient way to fulfil our duties towards humans (or other animals) [23]. We thus need arguments showing a relation between the desired outcome and the suggested research design, as well as arguments showing that there is a reasonable expectation of reaching the (direct or final) aim with the experiment [24]. This does not only hold for the justification of basic research [25], it equally is a crucial question for preclinical research. A growing number of publications show that the translational value of animal data is relatively low, i.e., the clinic does not mirror findings in animal experiments (cf. [5, 26, 27]). In the next sections, we focus on neuropsychiatry-related preclinical research as a case study to analyse the impact of the challenges of uncertainty and problems of translation on the ethical assessment.

### The relevance of the aim in preclinical research

The ability of an animal model to deliver valid results depends on, among others, adequacy of this model to simulate the phenomena under study, the reliability of the methods and experimental design, staff competence, the quality of the facilities used, and the communication of research results [2, 28]. High study quality is essential to the success of an animal experiment and therefore highly relevant for the ethical justification of any animal experiment.

Only experiments that are based on proper scientific reasoning and that use proper methods can deliver reliable results that can function as, metaphorically speaking, a brick in the cathedral of knowledge [29]. This can be understood as a procedural criterion in the discussion on animal research; the animal experiment is justified as long as it is conducted in a methodologically sound manner [3]. However, this criterion already includes a normative dimension. It starts in the recognition of the value of knowledge as such [30] and of the impossibility to predict future implications of any research. Based on these assumptions the procedural criterion can be understood as a sufficient condition to justify research that involves animals, because it ensures studies delivering reliable results. From this perspective, further considerations of the relevance of the research question are not necessary for the justification.

In contrast, others consider that animal use can only be justified if one can prove that a specific study has direct applicability for alleviating human or animal suffering [25]. As accepting research involving animals is only possible under specific ethical constraints, the relation between the direct and final aim and the used study design becomes pivotal in the question whether we should use animals in scientific research. These considerations are further complicated ‘when animals are used as models for humans, as the question of whether reliable extrapolations can actually be made from one species to the other, needs to be addressed’ ([3], p. XXI). Before analysing the probability that a study will lead to the aimed result, we need to distinguish between the direct and the final aim.

We define the direct aim as testing of the research hypothesis; e.g. introduction of the independent variable A causes a change in the measurable levels of dependent variable B. At this level, scientific scrutiny is essential. If we focus on the direct aim, one may consider an experiment as ethically acceptable if:

1) the experiment is conducted in a methodologically sound manner,

2) the chosen methodology can answer the research question,

3) the research question could not have been answered without the use of animals,

4) the number of animals used has been reduced to a minimum,

5) any unnecessary suffering of animals was prevented.

The first two points relate to the direct research aim. The remaining three points relate to the minimal ethical consideration on the use of animals: the 3Rs (replacement, reduction, refinement) [19].

We consider the final aim as the ultimate reason for conducting research activities, e.g. the pursuit of knowledge as a value in its own right or alleviating human suffering. In case of preclinical research of neuropsychiatric disorders, the final aim could be to gain knowledge on human neuropsychiatric disorders (or their selective symptoms) [2, 31, 32]. The ethical assessment of this ultimate aim requires realistically assessing the probability of reaching that final aim. This, however, is complicated, as it will depend on numerous conditions. The most important of these conditions is the translational validity of an animal model, i.e. its ability to accurately and sufficiently represent the condition under research [31]. Using models that are not valid is scientifically uninformative and morally unjustified [33]. However, it is difficult to obtain scientific agreement on the translational value of any given animal model (cf. [7]).

### Neuropsychiatry-related research: complexity and uncertainty

Uncertainty on the translational value of models is present in any field of research. However, the extent of uncertainty is especially high in preclinical research of neuropsychiatric disorders. In this section, we elaborate on this claim.

From the methodological perspective, animal models are not simply phenomenological copies of human phenotypes, they are rather complex theoretical constructs which require series of assumptions (e.g., about similarity of neurological systems or the importance of social behaviour). For neuropsychiatric disorders, animal models should be regarded as complex theories ‘about the aetiology and neuronal mediation of psychiatric disorders’ [31]. Consequently, estimation of the validity and reliability of any animal model benefits from ‘a sound theory about the disorder and the related theories underlying the model’ [31]. This condition is hard to fulfil for animal models of neuropsychiatric disorders, because neuroscience struggles to create coherent and comprehensive theories on neuropsychiatric disorders at various levels of scientific conceptualization. First, the etiology of neuropsychiatric disorders is poorly known, multifactorial “and/or there is an inability to alter the known etiology of a particular disorder” [34]. Second, there is a lack of knowledge of pathophysiology of neuropsychiatric disorders [35]. Third, theories on the etiology of neuropsychiatric disorders are difficult to falsify as it is hardly ever possible to conduct controlled experiments on human subject. Therefore, it is virtually impossible to distinguish between risk factors, triggering factors and resulting symptoms in human studies. Finally, neuropsychiatric disorders are defined by a list of symptoms out of which only some need to be present to diagnose a patient [35]. However, the symptoms that are not necessarily present in all the patients are often considered as necessary for the validity of new animal models [31]. Besides, many symptoms that are part of a neuropsychiatric diagnosis are subjective and perspective-dependent [36]. While patients can report on their emotional status, subjective symptoms cannot be modelled reliably and accurately in animals, thus raising concerns on model validity.

These methodological and conceptual difficulties are recognized. However, they are rarely discussed within the scientific community [2, 37, 38]. The awareness of limitations in current knowledge of the etiology and pathophysiology of neuropsychiatric disorders is even used as an argument to emphasise the importance of conducting animal research. It is argued that because it is virtually impossible to perform controlled experiments of risk factors in humans, and because the etiology is unknown, we are required to use animal models to fulfil our duties towards patients. However, one could equally use the shortage of clinical knowledge as a strong argument to restrict the use of animal models for neuropsychiatric disorders, because the lack of knowledge and falsifiable theories hampers the establishment of models with construct validity [23, 24, 38]. The lack of knowledge on ethiology and pathophysiology also undermine the results obtained from the currently used animal models of neuropsychiatric disease [37, 38].

One could still argue that the complexity and related uncertainty described above is not exclusive to neuropsychiatry-related research but that it is an inherent feature of any study using animal models. In biology a considerable continuity in biological (including genetic), anatomical, physiological, neurological, biochemical and pharmacological properties is assumed between animals and humans. If this assumption is true, one can agree that it is possible to e.g. study the dopaminergic system in a mouse brain (e.g. [39, 40]) as an approximation of the dopaminergic system in a human brain. In this type of mechanistic research, it is not necessary to postulate that the animal model is a model of a human disorder. Instead, it is a model of human neuroanatomy or biochemistry. However, even this simple logical construct can be questioned. Indiscriminate acceptance of this continuity can be criticised by pointing out differences, and by erroneous predictions based on animal models [24, 41, 42].

We do not consider it helpful for the ethical or the scientific debate to frame the discussion on this debate in an either-or dichotomy, since this often ends in a deadlock that neither improves scientific quality nor the position of the animals. For our current aim, it is not necessary to discuss the validity of animal models in general [1]. Rather, the validity of any given animal model needs to be evaluated in relation to the specific direct and final research aim [38]. That condition results in some problems that are specific to neuropsychiatry-related research.

The example of the dopaminergic system describes research of neurobiological processes that are postulated to underlie the pathophysiology of neuropsychiatric illness. This type of animal experimentation is not using an animal model of a neuropsychiatric disorder sensu stricto. The understanding of what an animal model for a neuropsychiatric disorder is, changed over time. According to the previously popular approach, an animal model is valid if and only if it resembles all the symptoms of a given disorder. This method is however losing its support in the scientific community as it becomes obvious that no animal model manages to mimic all aspects of a disorder. Besides, this approach requires assuming that it is possible to evoke states that are comparable to human depression or psychosis in animals. This assumption cannot be tested.

The current approach to preclinical research of neuropsychiatric disorders requires that an animal model resembles part of a psychiatric disorder, e.g., behavioural, cognitive or emotional phenotype [43]. The resemblance is evaluated based on face validity. This approach can be criticized in two ways. First, it requires the assumption that human and animal experiences are comparable in nature, which was elegantly refuted by Thomas Nagel [44]. Second, despite the similarities between animals and humans, there is no guarantee that the same mechanism underlies phenotypes that are related to each other based on face validity alone [38]. As Nestler and Hyman [38] express it: ‘There is an important chasm between the claim that disruption of some biochemical pathway regulates behaviour and the claim that it models a particular human disorder with useful implications for pathophysiology or treatment development. According to the ‘behavioral common path’ [45], multiple biological processes take place within the organism which may eventually be reflected in a limited repertoire of behaviours. Therefore, it is not possible to speculate which biological mechanisms underlie the phenotype under observation [45]. From this perspective, the probability of deducting the biological basis of behavioural manifestations of human neuropsychiatric disorder is low when animal models are based on face validity for the human phenotype under study. These criticisms add an additional level of uncertainty to the use of animal as models of neuropsychiatric disorders.

The uncertainty that is especially present in neuropsychiatry-related preclinical research complicates an ethical evaluation of the use of animals for this field of research. Although the societal relevance of alleviating health and welfare problems related to human neuropsychiatric illness will be commonly acknowledged, the uncertainties with regard to the validity of the models hampers the possible justification of using animals for this type of research. Therefore, reflection on the uncertainties is essential. On the one hand, it is important from an ethical perspective since it touches upon broader questions of how much uncertainty is allowed in ethical reasoning and the conditions of precautionary reasoning (e.g., [46]). On the other hand, it is essential because if this aspect is ignored it may lead to the use of models with only face validity to answer research questions requiring models with construct validity [31, 38]. In contemporary research, a lack of models with construct or predictive validity results in the use of models with only face validity [38]. Furthermore, reflection regarding clinical facts and the theoretical basis of models is lacking. This leads to a situation in which validity is assumed based on the amount of publications using a certain model or on the lack of other models [38]. However, this practice does not correspond with the final and direct aims of research. Continuing the use of animal models that lack construct validity may result in weak translational value and poor predictive power for drug effectiveness [47]. Ultimately this could result in a virtual “standstill” in the process of discovering new psychiatric drugs [38] accompanied by the unnecessary use of animals for research [2].

To summarise, a proper ethical evaluation of animal use in neuropsychiatry-related preclinical research is complicated by high levels of uncertainty. Although uncertainty is an inherent part on any scientific endeavour, it elicits specific questions for research on the biological basis of neuropsychiatric disorders. This is the result of our limited knowledge on the human neuropsychiatric illnesses which are being modelled. Consequently, the translational value of some of the currently used animal models may be difficult to prove, but also to debunk, which results in an ethical problem regarding the justification when using these models.

### Research on anorexia nervosa (AN) as a case study

We would like to illustrate the above-mentioned situation with a case study. We focus on anorexia nervosa (AN) and the preclinical research on one of its symptoms: high levels of physical activity, which for the purpose of this paper will be called excessive exercise. We selected AN out of personal interest and experience of one of the authors with AN animal models [48, 49]. Besides, while the main characteristic of anorexia nervosa, intense fear of gaining weight, cannot be analysed in animal models, several of the symptoms (reduced energy intake and weight loss) can be assessed objectively. Moreover, the ethics of animal models for several other disorders (e.g. schizophrenia, depression, ALS, neuropathic pain and OCD) have previously been discussed ([36]; Vieira de Castro and Olsson, 2014), while the ethics of AN models has to the best of our knowledge not been specifically assessed before.

Excessive exercise, in combination with other factors, can contribute to the development of AN by facilitating body weight loss [50]. From this perspective, research on excessive exercise has high clinical relevance, and various experiments tried to establish animal models of this condition. However, several unresolved issues exist on the exact nature and role of excessive exercise in the etiology of AN. First, there is no clear definition of excessive exercise in AN [50–54]. Consequently, it is not possible to create an animal model of excessive exercise with accurate face validity. Second, excessive exercise is only a single aspect of AN, which is neither required nor sufficient for diagnosis [55]. Third, excessive exercise in AN may be related more to co-morbid disorders than to AN itself; there is e.g. a positive relationship between obsessive-compulsive disorder and excessive exercise in patients with AN [56]. Fourth, it is unclear if excessive exercise should decrease during the treatment of AN [57–59]. As animal models are often used to screen for new treatments, the value of an animal model of excessive exercise in AN cannot be established. If one cannot expect a decrease of excessive exercise as a consequence of successful treatment, one cannot use it as a behavioural readout for a pharmacological screening test. One could ask whether we should strive to discover a pharmacological treatment for excessive exercise in the first place as a behavioural intervention may be more appropriate. Fifth, it is not established whether excessive exercise is a premorbid characteristic of AN patients [53, 59] or whether it is evoked by an extreme food restriction [60–63]. Despite the aetiology not being resolved, animal models of excessive exercise in AN have been created. We will further focus on the so-called activity-based anorexia model (ABA). The ABA model is considered the most promising animal model of AN because of its apparent face, construct and predictive validity [64, 65] and one paper even states that it is probably the best animal model within all animal models of human psychiatric illnesses [66]. Strictly speaking ABA is not a model for AN as a whole, but only for one of its symptoms, namely excessive exercise evoked by food restriction and body weight loss. In the ABA, restricted feeding results in high wheel running activity levels, which lead to a further reduction in body weight and food intake. However, the assumption that the excessive exercise seen in AN is merely a result of body weight loss has not always been confirmed in human research [53, 57]. The ABA model was established based on a theory of one specific aetiology of excessive exercise in AN, which was not confirmed in clinical research. If this aetiological theory is correct, the ABA model possesses apparent construct validity. However, one could argue that the face validity of the model is limited at best. It thus is hard to predict to what extent the ABA model could be used to unravel the neurobiological basis of excessive exercise in AN.

Despite the above-mentioned concerns on the use of excessive exercise as a read-out of clinical improvement, the ABA model has been used to test various neuroactive compounds with the aim of finding substances that can decrease the excessive exercise and increase body weight. However, we note that the translational value of the ABA model can be questioned on the basis of available data [66]. Although various compounds (targeting various brain systems, e.g. dopaminergic, serotonergic, melanocortinergic and opioid systems) decreased activity in the ABA, they were not effective in AN patient treatment [66]. Furthermore, while leptin levels correlate with physical activity in AN-patients [67] and leptin injections diminish running wheel activity in the ABA model [68], these injections also decrease food intake even further and pose a threat to body weight restoration [69, 70]. Therefore, one may conclude that the ABA model has limited use in testing compounds which could be used for symptomatic treatment of AN [66]. Moreover, the ABA model is based on the assumption that it is possible to compare excessive exercise in humans with a specific form of hyperactivity measured in rodents: high running wheel activity. Given the uncertainty of the translational value of the model, this also adds to be careful in choosing the ABA model to answer specific research questions.

These concerns and the conclusion about validity and translatability are not restricted to the ABA model. It also applies to other animal models. Therefore, the challenges cannot be addressed by just choosing another model. The discussion of the ABA model shows general challenges that the field of neuropsychiatry preclinical research is facing. Therefore, the importance of the case study is not limited to the discussed model.

### Three possible steps to change and combine science and ethics

The importance of questioning the validity and translational value of animal models is recognized by researchers who strive to improve the existing situation. Proposed solutions target this issue at three levels.

First, to increase reproducibility of results, there are attempts to improve the methodology. That can be pursued in different ways. On the one hand, one can aim to standardize tests between different laboratories, while incorporating standardized variation in the experimental designs to increase external validity. A recent example of this approach is described by Grandjean et al. [71], who standardised the fMRI analyses for a multi-centre mouse study, maintaining cross-laboratory differences in equipment and procedures. Standardisation between laboratories increases the ability of the animal model to reach the direct aim and reliably address the research hypothesis. However, without external model validity it has only limited effects on reaching the final aim of research if the ultimate reason for conducting a specific research activity is finding an effective treatment for humans. On the other hand, the use of Systematic Reviews (SRs), i.e., in-depth analysis of previously performed experiments, can be essential to increase research quality, and to maximise use of the available data [72, 73]. This will not solve the validity problem as such [74] but can help to trace pitfalls and provide evidence about the (lack of) translational value of animal models [75] and enable to estimate the evidential weight of animal models [76–78].

Second, it is important to acknowledge that an experimental animal is not a patient. That may seem a truism. However, research models always have to balance between the clinical heterogeneity due to the complexity of the individual patients and the need to test with standardised animals under standardised conditions. The arguments of feasibility and replicability lead to a demand for standardisation, whereas successful translation to the variety of patients asks for incorporation of complexity and diversity. This can be called the “standardisation-translation paradox” [79]. To tackle this paradox, it is essential to start designing research models incorporating the complexity of the patient, including e.g. specific genotypes and personal histories. Animal models should reflect the variation between patients to increase external validity; testing should be performed in e.g. young and old animals of both sexes with different genetic backgrounds as far as these characteristics are relevant to the patient population. Note that while we encourage increasing the complexity of the modelled *patient*, we do see potential value in reducing the complexity of the modelled *pathological process* and assessing endophenotypes reflecting only part of a complex disease, as advocated by e.g. Cryan and Slattery [80]. Tackling the standardisation-translation paradox also requires reversed translation: the research question should be formulated from a clinical context and then translated into a specific question that can be addressed with an (animal) experiment. In our experience, many animal studies start with a question that may seem clinically relevant and start with an existing animal model that has been used in the laboratory before. Furthermore, tackling this paradox asks for standardised variation. To mimic the patient in preclinical research we have to incorporate the variation we find in the patients into our research in a standardized manner. Relevant variation (e.g., gender, genetic background) has to be incorporated into preclinical research. In practice, this recognition of clinical heterogeneity within mental disorders and their comorbidities caused a shift from modelling mental illness to modelling phenotypes. Validity and translational value need to be examined for each phenotypic model to the same extent as before for the ‘full disease models’. This requires that scientists in the field of neuropsychiatric disorders more honestly assess the potential benefits of their research efforts a priori. This task is challenging, but necessary to ethically justify the use of animal experimentation. Furthermore, while it is not as commonly performed by ethical review boards as we would have hoped (Vieira de Castro and Olsson, 2014), it is possible. There are guidelines to aid estimating the possible benefits and harms of the use of any given animal model, for example the guidelines proposed by the Federation of European Laboratory Animal Science Associations ([28], Table 2). However, the available guidelines hardly address the question of transferring knowledge across species, which should, in our opinion, be added to allow for honest assessment of the potential benefits and harms.

Third, more collaboration in and integration of the research chain is needed. If innovation with respect to validity and translation remains at the level of individual research groups, not much will happen. This is not due to incompetence or indifference, but the development of new models is not an easy task and in practice easily hampered by processes within the scientific community. Creating a new animal (free) model is a time consuming and unrewarding task. It is challenging to validate a new model to the extent that satisfies the scientific community and the legal guidelines. Therefore, researchers preferably use established animal models, even if their validity is limited. This strategy maintains *status quo* and discourages creative solutions. Creating a new animal (free) model with good translational value and validity is further complicated by the limited knowledge of etiology and pathophysiology of neuropsychiatric disorders discussed above. This shows the need of more consorted action. It requires improved cooperation between clinical and preclinical researchers, but also journals and legislators. Although the expertise to innovate is at the level of the individual researchers, the responsibility is shared and cannot be limited to this group of people.

Additionally, addressing problems of validity and translation requires input from various disciplines. Given the transdisciplinary nature of current research consortia this may not seem a real challenge. However, difference in the basic assumptions between science and ethics may seriously complicate model development in the field of neuropsychiatric disorders in three ways. Firstly, scientists conducting (neuro)-psychiatric clinical and preclinical research do not share a single view on the nature of neuropsychiatric disorders; they have different implicit and explicit biological, anthropological and philosophical theories on disease pathology, causality and the mind-body dualism. This hampers collaboration and efforts of creating clear guidelines for preclinical research. Furthermore, not all neuropsychiatry-related preclinical research has the alleviation of human suffering as its final aim, the aim may be restricted to advancing knowledge. For example, it could aim to understand neuronal systems which might be involved in neuropsychiatric disorders and testing of new therapeutic agents [2].

Finally, also for researchers in this field holds that there is a fundamental plurality of views on the moral status of animals and the relevance of ethics. This combination creates a complex matrix of possible positions in the ethical justification, recognition of ethical dilemmas and scope of solutions that are considered acceptable. It requires a level of reflection and openness to the normative and scientific assumptions that goes beyond the old-fashioned views of ethical dilemmas as subjective [81] and irresolvable. We agree with Gluck and Bell [23] that researchers cannot leave consideration of the moral dilemmas to others, as this may lead to scientific practice based on “questionable prejudices”. We consider it to be the professional responsibility of researchers to work on the translatability of results, and to consider the ethical dilemmas resulting from epistemological uncertainties. This is not only a theoretical ‘ought’; recent examples (cf. [82]) show that it is possible to combine the preclinical and clinical context with attention to the ethical dimension to discuss the translational neuroscience.

It is important to stress that from this perspective, ethical reflection is not an add-on to the science debate, but research quality benefits from ethical reflection. This, however starts from the notion that ethics in the context of animal research cannot be reduced to the ethical principles of reducing harm and applying the 3Rs. These two are important, but insufficient principles to discuss the quality of the used models and to explore innovative research methods. Ethical deliberation in the context of animal includes also the principles of transparency and consistency. This means, for instance, that the steps in the process of choosing a research model must be verifiable and open to discussion with an interdisciplinary group of peers. Furthermore, ethical reflection enables to deal with problems of uncertainty and the evaluation of benefits (cf. [83]. This is not only relevant in the ethical assessment in the context of a harm-benefit analysis, but also in defining what model should be used for what aim. The choice of a research model comes with questions of uncertainty and probability about interspecies comparison and linked to views on the direct and final aim of the research. Both dimensions include a normative component that requires ethical reflection (cf. [4, 84].). Therefore, ethics in the context of animal research should have wider focus than on animal protection only and can in this way contribute to the quality of research.

## Conclusion

The aim of this paper was to present and analyse animal use for preclinical research on neuropsychiatric disorders as a moral problem which demands combined ethical reasoning and logical evaluation of scientific practice. We conclude that this moral problem is specifically complicated in neuropsychiatry-related research, due to the limited knowledge on neuropsychiatric disorders in humans and resulting in difficulties with establishing valid animal models for these disorders. Consequently, preclinical research is characterized by the frequent use of animal models which do not possesses sufficient validity to reach the direct or final aim of research. This raises an ethical concern, because current policies require that ethical justification presumes the probable gains for humans outweighing the suffering experienced by animals. The problem of translation frustrates this justification. Therefore, we proposed three steps to address the problems of validity and translation: optimising the methods, incorporating the complexity of the patients into the models, and increased and collaboration within the research chain. This entails a scrupulous analysis of currently used animal models to improve the applicability of research. In this process systematic reviews can provide relevant information. Furthermore, it is essential to start with the clinical heterogeneity and design research models that are better capable to mimic the complexity of the patient. This requires reversed translation: the research question should be formulated from a clinical context and then translated into a specific question that can be addressed with an (animal) experiment, rather than to start with the existing animal models as the golden standard. This task is challenging, but necessary to ethically justify the use of animal.

Finally, we proposed that the previous steps require more and better cooperation between partners in the research chain from bench to bedside and between the relevant disciplines. This is not only a matter of improved organization and procedures, but also of attitude. Innovation that leads to enhanced validity and translation of models used in neuropsychiatry-related preclinical research is only possible in an open dialogue about the aims of the research and the relevant models, in awareness of the plurality of views on both the scientific and ethical level. For ethics this entails that ethics in the context of animal research cannot be reduced to the ethical principles of reducing harm and applying the 3Rs. To contribute to the quality of animal research is equally should deal with broader issues such as uncertainty, evaluation of benefits and transparency. The combination of ethics and science in this discussion is not a detour but helps to get a grip on the complexity of the issues at stake. It can improve the clarity of the discussion by helping to distinguish between questions that have their origin in ethical viewpoints from those that relate to the scientific validity of the models. Furthermore, it helps to combine firm knowledge of human psychiatric disorders with the relevant values and interest at stake to come to an honest evaluation of currently available preclinical models. It is important that national and international research societies put this combined approach to the challenge of translation on the agenda more prominently.

## Data Availability

Not applicable.

## Abbreviations

3R: Principles of replacement, reduction, refinement
ABA: Activity-based anorexia
AN: Anorexia Nervosa
HBA: Harm-benefit analysis
SR: Systematic Review
